# Supplementary material for: Clustering knowledge and dispersing abilities enhances collective problem solving in a network
Source: Nat Commun. 2019 Nov 13;10:5146. doi: 10.1038/s41467-019-12650-3 (PMC6853876; doi:10.1038/s41467-019-12650-3)

Clustering knowledge and dispersing abilities enhance collective problem solving in a network.

Gomez and Lazer

Supplementary Methods

We first test whether having more than two species of agents in the diversity of ability simulations might result in a different outcome. We reran our core results in the NK space with five species of agents: Species 1, Species 2, Species 3, Species 4, and Species 5. Just as with the A/B setup, there is nothing inherent about a Species 1 agent other than it is a nomenclature meant to signal that an agent has a mutually exclusive search heuristic from the other four species. The heuristics used by each species of agents is uniquely assigned for each simulation. In other words, as there are four types of heuristics and a mask that can include any of the N=20 bits, the combination of these two search criteria ensures that no two heuristics are similar in any given simulation. However, we hold the initial states of knowledge and species constant across the five *intermixing* distributions to compare them. These *intermixing* rates follow the five distribution types for the diversity of simulation models, which use five states of knowledge instead of five species of agents. As such, and following a similar logic to the diversity of knowledge simulations, blocs of agents are randomly interspersed so that full *intermixing* is, in fact, the random *intermixing* of the five species of agents. Subsequent distributions of these "agent-blocs" are randomly distributed such that blocs of 10 "Species 1" agents may be next to a bloc of 10 "Species 3" or "Species 4" agents instead of always being next to 2 and 5.

Supplementary Figure-1(a), Supplementary Figure-1(b), and Supplementary Figure-1(c) are set up in a similar format as Figure 4(a), 4(b) and Figure 5 in the main text, respectively, but now plot the simulation results with five diverse agents instead of two. (To highlight the

differences in performance, we raise the NK scores here to the 5th power instead of 10th as was done in the main text to accentuate the differences in performance.) We find that our core results still hold with five diverse agents.

We then show that similar results hold across various manifestations of a torus network, like Watts-Strogatz's small world model[1]. Increasing the "small world" effect shifts the mechanism driving the system to more exploitation over exploration. We use three small world settings based on the number of rewires: a torus network with no rewires; a torus network with 30 links randomly rewired; and a torus network with 100 links randomly rewired. The networks are created before the simulations run and are held constant across each experiment. Second, if communication between agents in the network did not result in perfect transmission of ideas, we still ought to show the evolution of these results under these various conditions. Thus, along with network structure, we also implement a complex contagion parameter in our model to test the robustness of preserving the benefits of intermixing inspired by Centola and Macy[2]. Third, robust results ought to also hold in a different problem space with a different landscape and geography for agents to explore. To that end, we rerun all of our main results and the aforementioned robustness checks in a second problem space called the Traveling Salesperson Problem (TSP). Finally, we introduce a new form of diversity called A+ agents that encompasses the A/B species heuristics and search capabilities.

Our results ought to be robust and hold in a different problem space with a different landscape and geography for agents to explore. We rerun all of our main results and the aforementioned robustness checks in a second problem space called the Traveling Salesperson Problem (TSP). The TSP is a canonical optimization problem in computer science, because it becomes computationally impossible to solve this problem through brute force. In this problem, a

salesperson is presented with a series of cities they need to visit once, and they seek an itinerary that minimizes their total round-trip distance. While the TSP problem space is used in other domains to study complex parallel problem solving[3], it has been used far less than the NK space as a canonical model (although see Lawler, et al.[4]). The TSP problem space is comparable to the NK problem space because they are both partially decomposable to sub-problems that lead to globally optimal states of knowledge. However, the TSP problem space is less rigid than the NK space. In other words, the TSP space mirrors the NK space in Figure 1 in the main text, with a low value of K (i.e., a singular, large mountaintop, rather than a range of peaks and valleys). Agents in the TSP space all eventually ascend towards a global optimum, which is less likely the case in our NK space setup. To address this issue, TSP simulations are ended just before they reach an equilibrium and coalesce on the same global optimum. Nevertheless, our objective in using both TSP and NK problem spaces is to assess the robustness of our findings.

We define a TSP space based on a "map" containing twelve cities. The distances between cities are asymmetric, so the distance from city 1 to city 2 is different from the distance from city 2 to city 1. The asymmetry allows for more states of knowledge and widens the search space of the agents. At the start of each simulation, a unique TSP space was defined as a 12 x 12 matrix, where the rows are the starting cities, the columns are the next city traveled to from the starting city, and the entries are the distances from the starting city to this next city. At the start of each simulation random integer values between 1 and 100, inclusive, were calculated for every non-diagonal entry. In other words, the ijth entry in the matrix represents the distance from city i to city j, where city i is the ith row and city j is the jth column in the matrix. Alternatively, the jith entry represents the distance from city j to city i, where city j is the jth row and city i is the ith column in the matrix.

An agent receives an itinerary as an array of *n* numbers representing each of the cities. The goal of the TSP is to minimize the distance of one complete round trip without returning to any city already visited on the trip. The round-trip distance is calculated as follows:

$$Distance = ij + jk + kl + ... + xy + yz + zi \quad (1)$$

If city *i* is the 1st entry in the array, and the 2nd entry is city *j,* then the distance traveled is contained in entry, *ij*. Next, if city *j* is the 2nd entry in the array, and the 3rd entry is city *k,* then the distance traveled is contained in entry, *jk*. From the 1st to the 2nd city and from the 2nd to the 3rd, this process is repeated until the *n*th city is reached, where the final leg of the round-trip is computed as the distance from the *n*th city back to the 1st city. Each of these individual legs is summed as the round-trip distance for that particular itinerary.

In the TSP problem space, a given state of knowledge is represented as an ordered list of 12 cities in an itinerary, where every city appears once. The objective is to find the shortest round-trip, visiting each city at least once. Here, the same setup holds, A and B agents are randomly assigned a non-overlapping search heuristic unique to each problem space, but held constant across all five intermixing distributions. The heuristics that agents use are similar to the setups used in the NK space, but with some slight alterations to accommodate the different structure between the problem spaces. As the TSP space is more limiting in terms of how states of knowledge can be manipulated (e.g., an agent cannot turn a city to a 1 or 0 like in the NK space), agents in the TSP space can engage in one of four behaviors: (1) swap two adjacent cities in the itinerary (e.g., *city1* and *city2* or *city5* and *city6,* where *city1* refers to the first city in the itinerary, *city2* refers to the second, and so forth); (2) swap two cities that are not adjacent in the itinerary (e.g., *city1* and *city5* or *city2* and *city6*); (3) randomly jumble some number (less than *N*-2) of adjacent cities (e.g., *city3*, *city4*, *city5*, *city6*, and *city7*)*;* or (4) randomly jumble some number (less than *N*-2) of *non*-adjacent

cities (e.g., *city3*, *city6*, *city9*, and *city10*). For the latter heuristics that manipulate more than two cities, one agent is randomly assigned a mask that includes anywhere between 2 and 12 cities in the itinerary (i.e., state of knowledge), while the other agent is randomly assigned a mask that includes between 2 and 12 cities NOT previously assigned to the first agent. In other words, if agent A's mask involved manipulating *city2*, *city3*, and *city4*, then agent B is assigned any of the remaining cities (e.g., *city5*, *city6*, *city7*, and *city8*; or *city1*, *city6*, *city9*, and *city12*; etc.). Agents iterate the mask in a stepwise fashion such that agents adopt the state of knowledge (out of the N=12 possible ones) that has the best solution. For instance, agent A would jumble *city2*, *city3*, and *city4*, record its score; then shift the mask over by one city (i.e., *city3*, *city4*, and *city5*), record its score; and continue in a similar fashion until the agent has stepped through all twelve potential itineraries, subsequently adopting the optimal itinerary. This setup offers a direct parallel to the NK setup, and allows agents to avoid getting stuck at local optima.

For our diversity of ability experiments, how we define ability is subject to a wide-array of approaches to operationalize how it becomes implemented in either the TSP or the NK spaces. There are two ways that we address this. First, we test the opposite manifestation of mutually exclusive perspectives: namely, we run our experiments with agents whose perspectives are mutually nested within one another. Second, we tested a variety of mutually exclusive heuristics to ensure that no one heuristic was driving the results. In what follows, we outline both approaches in both spaces.

First, to test how opposite instantiation of mutually exclusive abilities, we define an A+ species agent that encompasses the search capability like those defined previously for the NK and TSP spaces. For instance, for the NK space search heuristics, agents are randomly assigned some mask and one of the four manipulation behaviors as previously outlined. Agents shift its mask

through all N=20 possible solutions and selects the best state of knowledge out of the twenty. The A+ agents, by contrast, adopt the same mask as the other agent for that particular problem space, however it shifts its mask using all four manipulation behaviors and selects the best possible state of knowledge. In that way, A+ agents encompass the entire search capabilities of the other agent in that A+ agents can see everything the other agent sees, plus more. Similarly in the TSP space, A+ agents apply all four manipulation behaviors, move in a stepwise fashion across all N=12 possible solutions for each behavior, and adopts the best possible solution produced, allowing the A+ agents to see everything the other agent sees plus much more.

We extend our torus network model by rewiring ties to mimic a small world model[1]. Along with network structure, we also implement a complex contagion parameter in our model to test the robustness of preserving systems with intermixing inspired by Centola and Macy[2]. We use three small world settings based on the number of rewires: a torus network with no rewires; a torus network with 30 links randomly rewired; and a torus network with 100 links randomly rewired. The networks are created before the simulations run and are held constant across each experiment.

We also built a complex contagion model (see Centola and Macy[2]) where an agent copies a set of knowledge if the agent sees it adopted by at least two of its neighbors. However, we slightly deviate from the Centola and Macy[2] model because allowing some slight diffusion of information from single adopters, arguably, has greater verisimilitude with actual diffusion processes, as Centola's[5] follow up experiment demonstrated. In our updated model, an agent can see a set of knowledge with 100 percent probability if it is adopted by at least two of its neighbors. However, there is some ε probability (where ε << 100 percent) that an agent will see the single state of knowledge of a solitary neighbor. And the assumption of zero diffusion without multiple exposures typically results in no diffusion at all when the system starts with 100 different agents.

We implement three degrees of contagion: simple, moderately complex, and complex. A simple contagion is the absence of the aforementioned criteria, whereby an agent can see all of its neighbors' states of knowledge each round with 100 percent probability. A moderate contagion adopts our above contagion model, where an agent can see any individual neighbor's state of knowledge with a 50 percent probability each round; but can see two or more neighbors' states of knowledge if at least two of them have the same state of knowledge. A complex contagion operates in the same way but reduces to a 10 percent probability.

Our robustness checks are run for both the NK and the TSP problem spaces and for both diversity of ability and diversity of knowledge experiments. We find that for both types of diversity of ability (B and A+), across contagion and network structure, increasing intermixing is associated with improved performance and these results across time.

As the problem spaces use two different scales to measure performance (e.g., NK is on a zero to one scale, while TSP is the mean distance between cities on a set grid), we need to render network performance comparable. As such, the average network performance is measured for each of the five intermixing setups across problem spaces separately at each round of the simulation. The network configuration (i.e., minimal, low, moderate, high, full intermixing, or random in the case of the diversity of knowledge simulations) with the best average result amongst its agents is counted as having the best solution for that particular problem space. In other words, this accounting for performance is setup as a winner-take-all or first-past-the-post approach: whichever intermixing setup found the best average solution in round *t* for a particular problem space, it "wins" this contest as having the best average solution among the five setups. As such, this setup also allows for two or more configurations to have settled on the same final average result, yielding "ties" among configurations having the best solution (and percentages that sum to mover 100%).

To that end, the *y*-axis captures the percent of these problem spaces that a particular network configuration found the best average result at equilibrium.

For the nested abilities as shown in Supplementary Figure 2, these results hold across problem spaces. (The Supplementary Figures presented here juxtapose the A and A+ results with the A and B results for comparison.) Since nested diversity setups have access to local optima, they have access to better states of knowledge than what is available to any exclusive diversity setup can find. Nested diversity setups are more likely to find the global optima. Thus, we expect to see a leveling-off of performance in these systems, because they are quickly finding global optima in proportion to the degree of intermixing. These results also hold over time, as illustrated in Supplementary Figure 3. And the number of unique sets of knowledge produced by these setups is similar to the A and B setups, as shown in Supplementary Figure 4.

Supplementary Figures 5(a) and 5(b) summarizes the results across the three different cases for contagion and rewiring, resulting in 9 distinct setups for the A and B setups, where each setup contains the results in both NK and TSP spaces. And Supplementary Figures 6(a) and 6(b) summarizes the results in a similar fashion for the A and A+ setups.

While rewiring naturally removes the benefits of intermixing (i.e., everything is randomly connected to distant parts of the network), increased contagion seems to not have much of an effect on the relative performance between setups with higher intermixing and those that do not. High contagion networks with stronger "small-world" setups seem to negate the effects of the rewiring. With more complex forms of contagion, or slower diffusion of knowledge, the system is driven more by exploration than exploitation. With more exploration, there are more opportunities to introduce more unique states of knowledge into the system. While we see that systems across the various distributions of diversity find optimal states of knowledge less frequently (i.e., fewer ties),

the performance differential between the distributions of diversity across the three types of contagion slightly attenuate with more complex forms of contagion. The benefits of intermixing are not reaped and fewer unique states of knowledge are introduced into the system. Thus, the performance differential between different rates of intermixing diminishes with more rewiring.

For the diversity of knowledge experiments, we see that our main results hold across problem spaces, as shown in Supplementary Figure 7, Supplementary Figure 8, and Supplementary Figure 9 in terms of final performance, progress over time, and the number of unique states of knowledge introduced into the system, respectively.

We also run these simulations along the different network and contagion conditions, as show in Figures Supplementary Figure 10 and Supplementary Figure 11. Similar to the diversity of ability experiments, our results broadly hold across these conditions. Much like the main results, minimal intermixing of states of knowledge helps to buffer against a cacophony of diverse states of knowledge, which, in turn, allows agents to uncover pathways to better states of knowledge. Higher degrees of small world-ness in the network undermine the buffer that non-intermixed states of knowledge afford, and the performance differential between intermixing systems attenuate. Just as with the diversity of ability experiments, more rewiring of the network prevents more unique states of knowledge to be introduced into the system.

Supplementary References

1. Watts, D. J. & Strogatz, S. Collective dynamics of 'small-world'networks. *Nature* **393**, 440–442 (1998).
2. Centola, D. & Macy, M. Complex Contagions and the Weakness of Long Ties. *Am. J. Sociol.* **113**, 702–734 (2007).
3. Dorigo, M. & Gambardella, L. M. Ant colonies for the travelling salesman problem. *Biosystems* **43**, 73–81 (1997).
4. Lawler, E. L., Lenstra, J. K., Kan, A. H. G. R. & Shmoys, D. B. *The Traveling Salesman Problem: A Guided Tour of Combinatorial Optimization (Wiley Series in Discrete Mathematics & Optimization)*. (Wiley, 1985).
5. Centola, D. The Spread of Behavior in an Online Social Network Experiment. *Science* **329**, 1194–1197 (2010).

Supplementary Figures

Supplementary Figure-1(a). The average NK score of networks across problem spaces, plotted by *intermixing* rates for the diversity of ability simulations with five diverse agents (NK Space). Error bars are the standard errors measured across NK spaces.

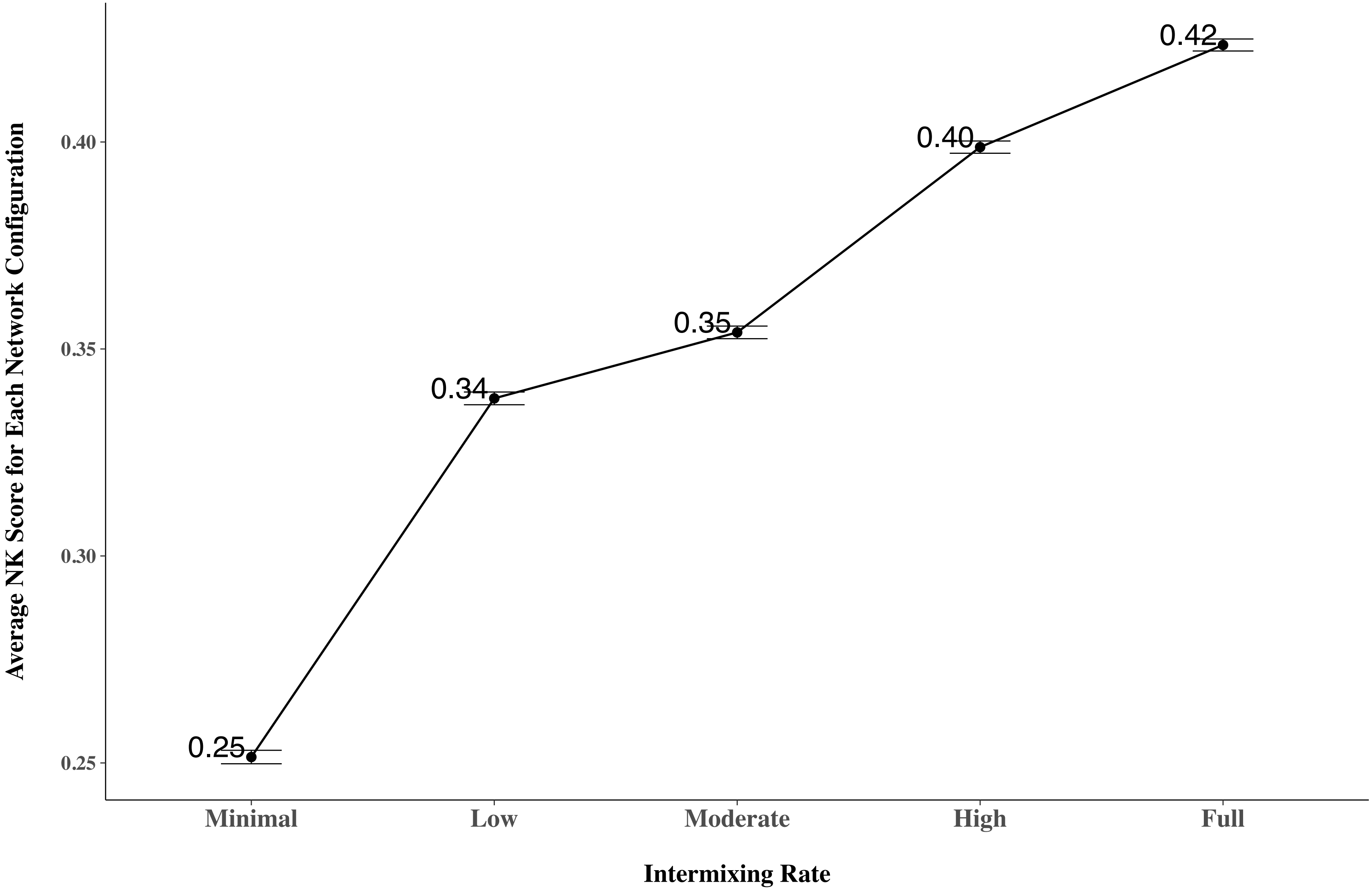

Supplementary Figure-1(b). The average NK score of networks across problem spaces, plotted over time for the diversity of ability simulations with five diverse agents by *intermixing* rates (NK Space). Error bars are the standard errors measured across NK spaces.

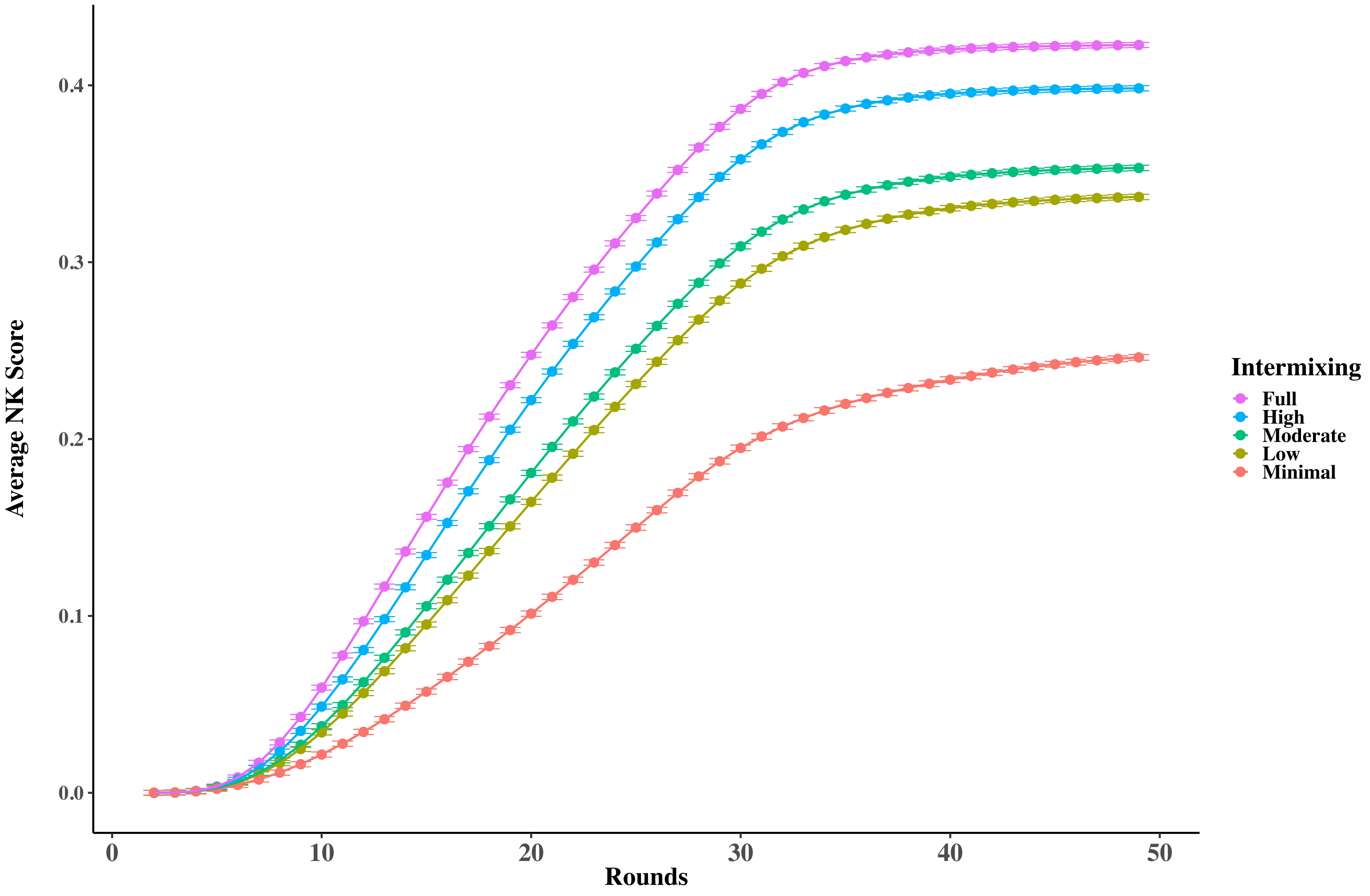

Supplementary Figure-1(c). The average marginal number of unique states of knowledge by *intermixing* rate over time for the diversity of ability simulations with five diverse agents, as compared to the number of unique solutions generated by minimal *intermixing* networks (NK Space). Error bars are the standard errors measured across NK spaces.

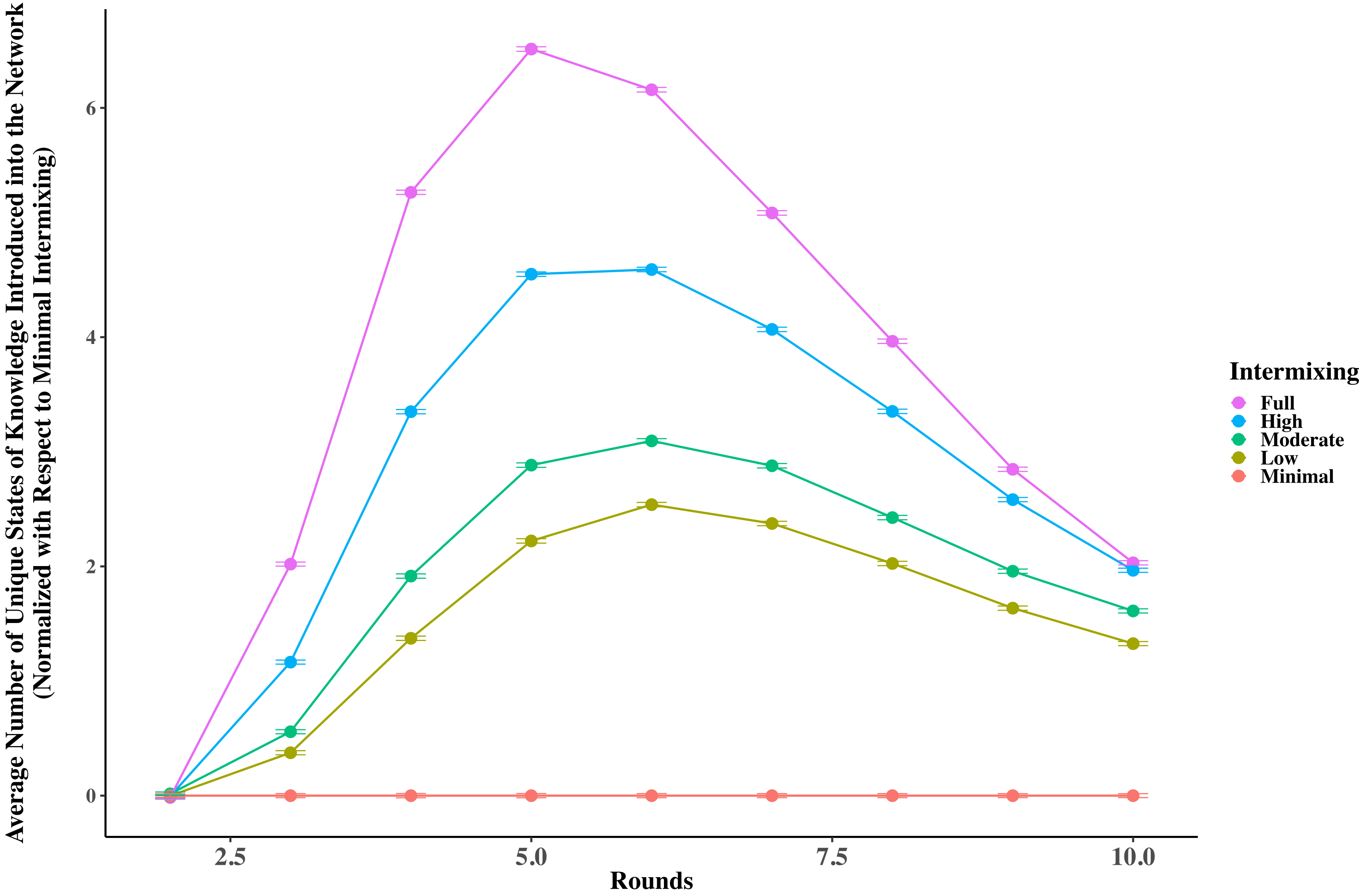

Supplementary Figure-2. Performance of networks with various intermixing rates for the diversity of ability simulations (NK and TSP spaces for A and B, A and A+).

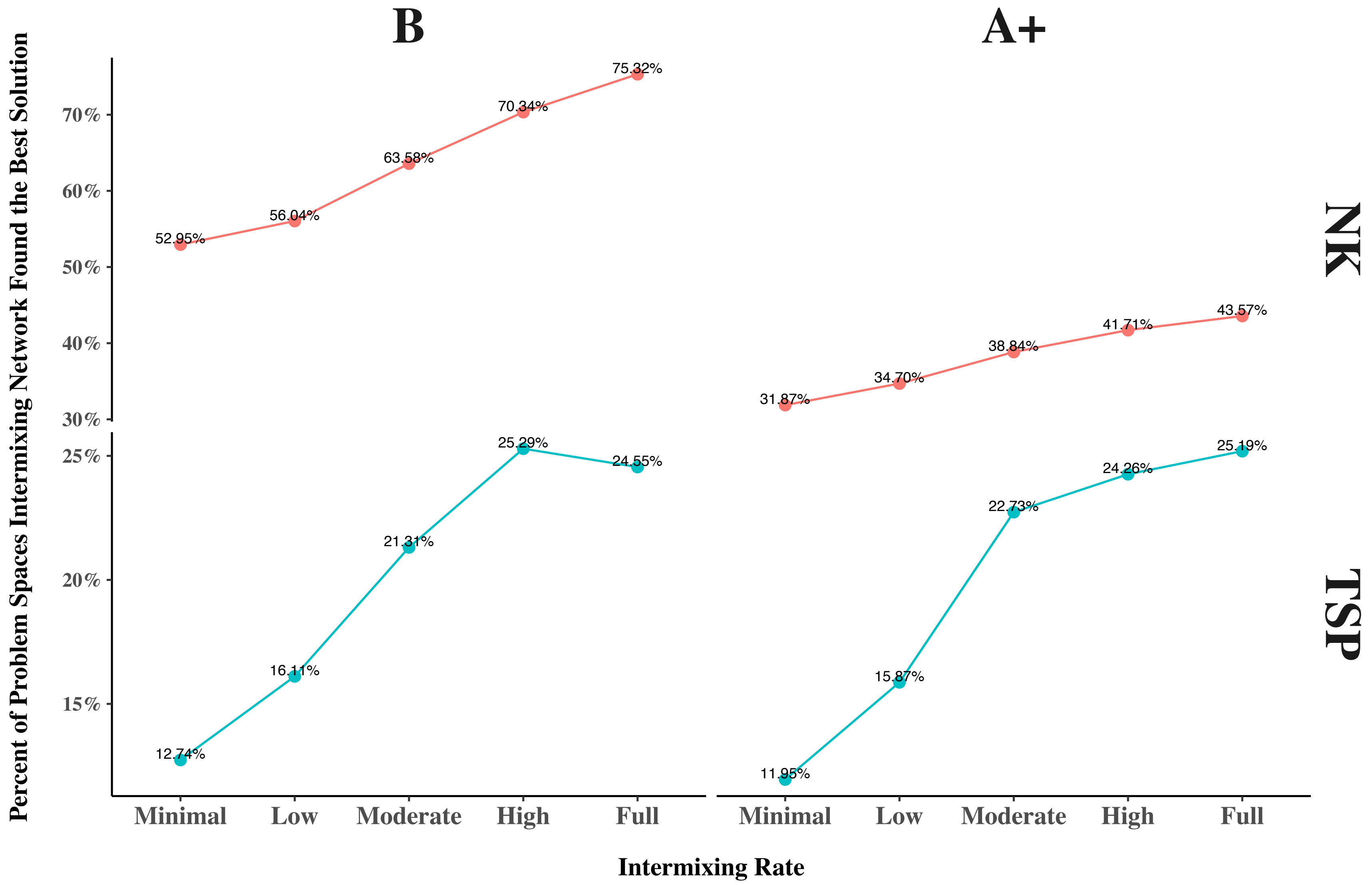

Supplementary Figure-3. Performance over time of networks with various intermixing rates for the diversity of ability simulations.

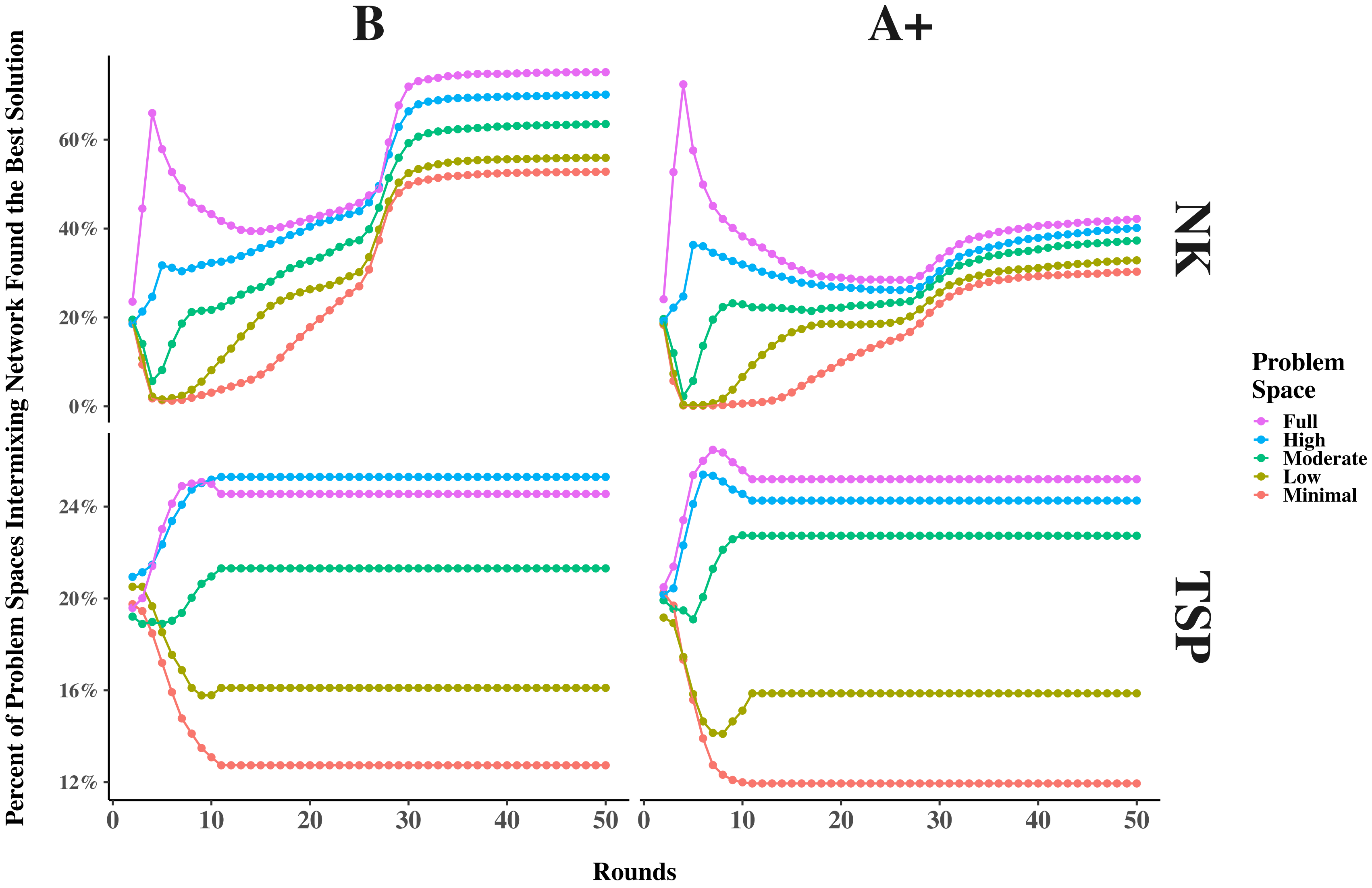

Supplementary Figure-4. Average number of unique states of knowledge by intermixing rate over time for the diversity of ability simulations. Error bars are the standard errors measured across NK and TSP spaces.

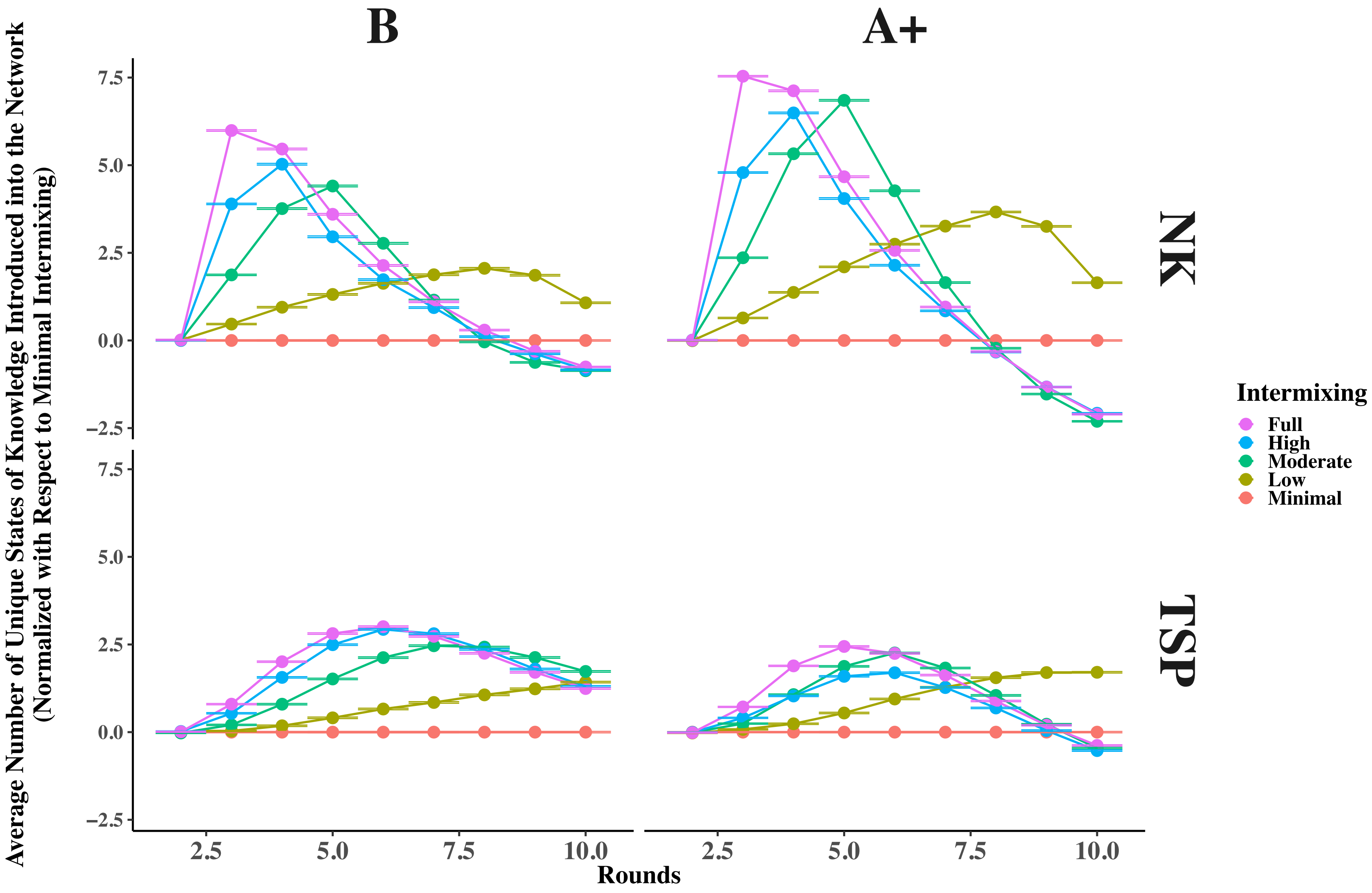

Supplementary Figure-5(a). Performance of networks with various intermixing rates for the diversity of ability simulations (small world and contagion setups, NK space for A and B).

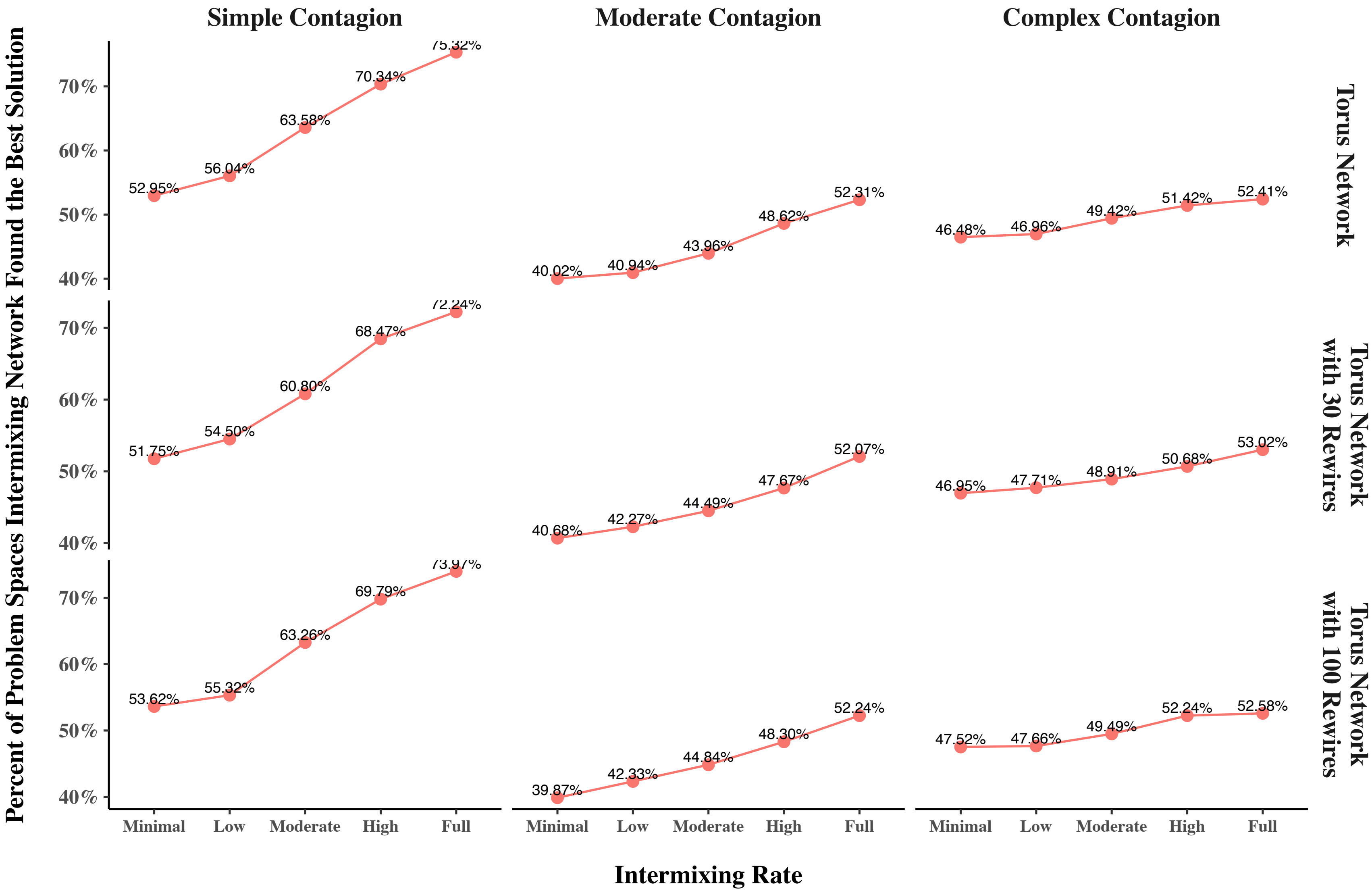

Supplementary Figure-5(b). Performance of networks with various intermixing rates for the diversity of ability simulations (small world and contagion setups, TSP space for A and B).

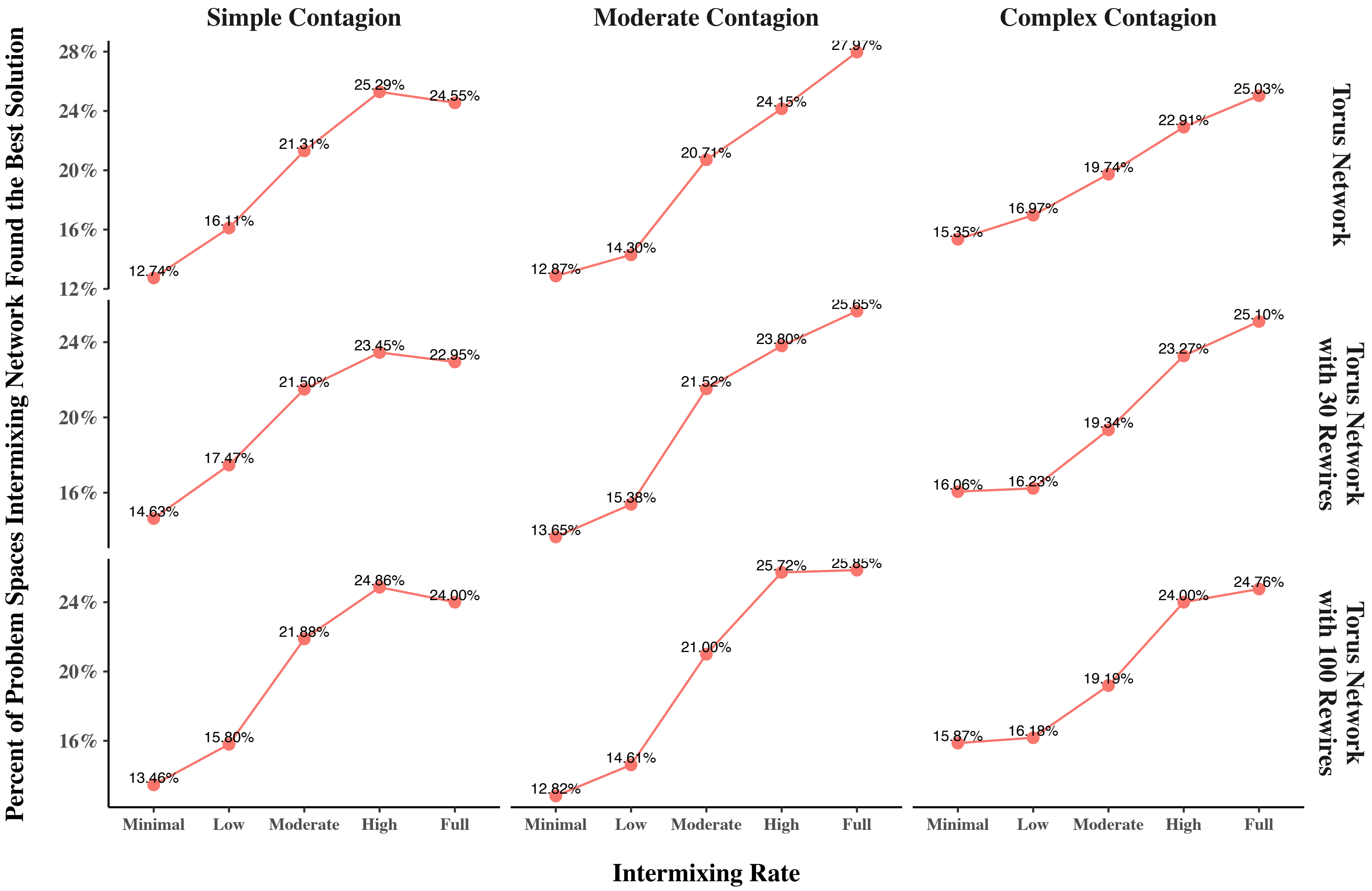

Supplementary Figure-6(a). Performance of networks with various intermixing rates for the diversity of ability simulations (small world and contagion setups, NK space for A and A+).

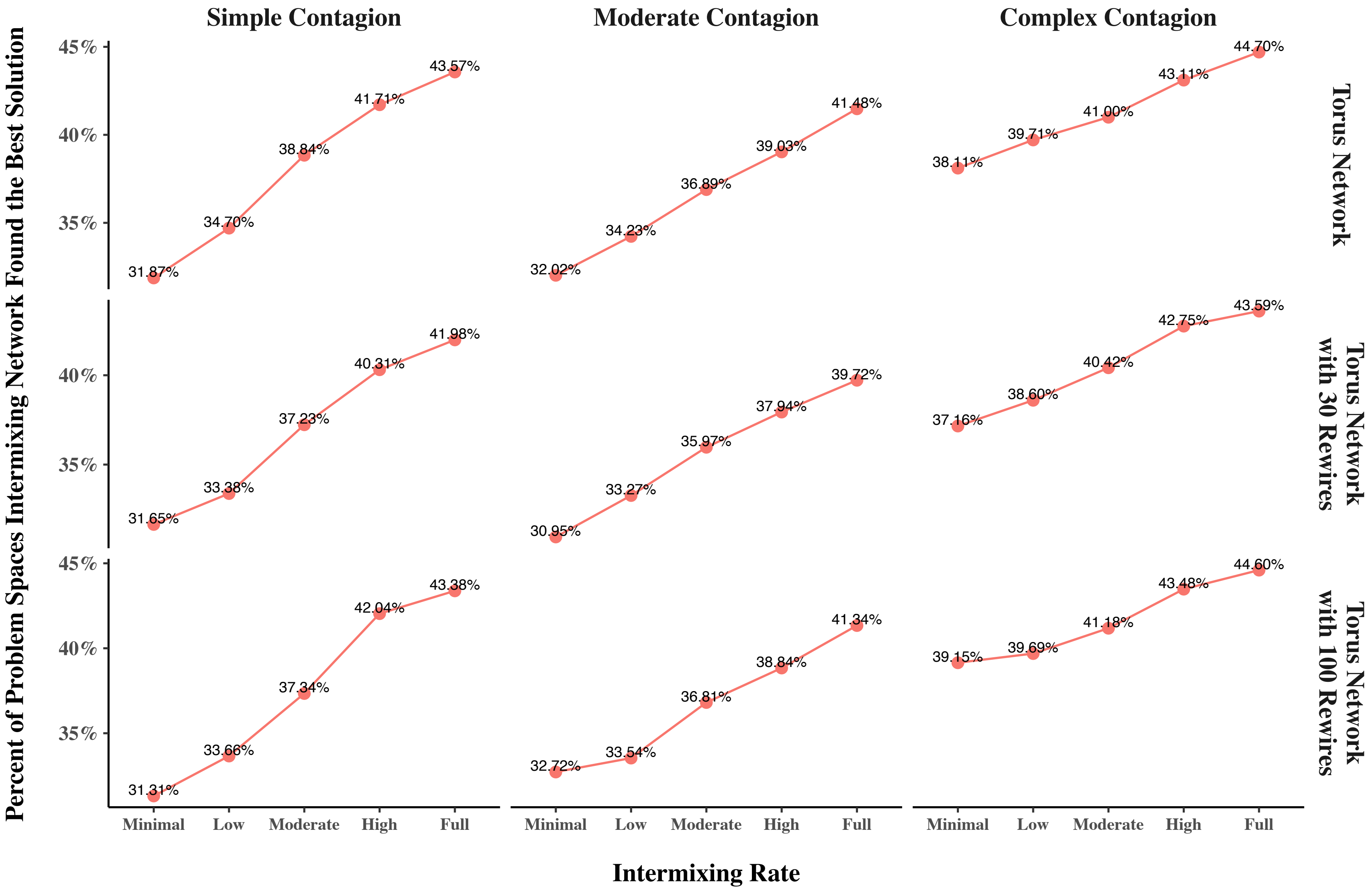

Supplementary Figure-6(b). Performance of networks with various intermixing rates for the diversity of ability simulations (small world and contagion setups, TSP space for A and A+).

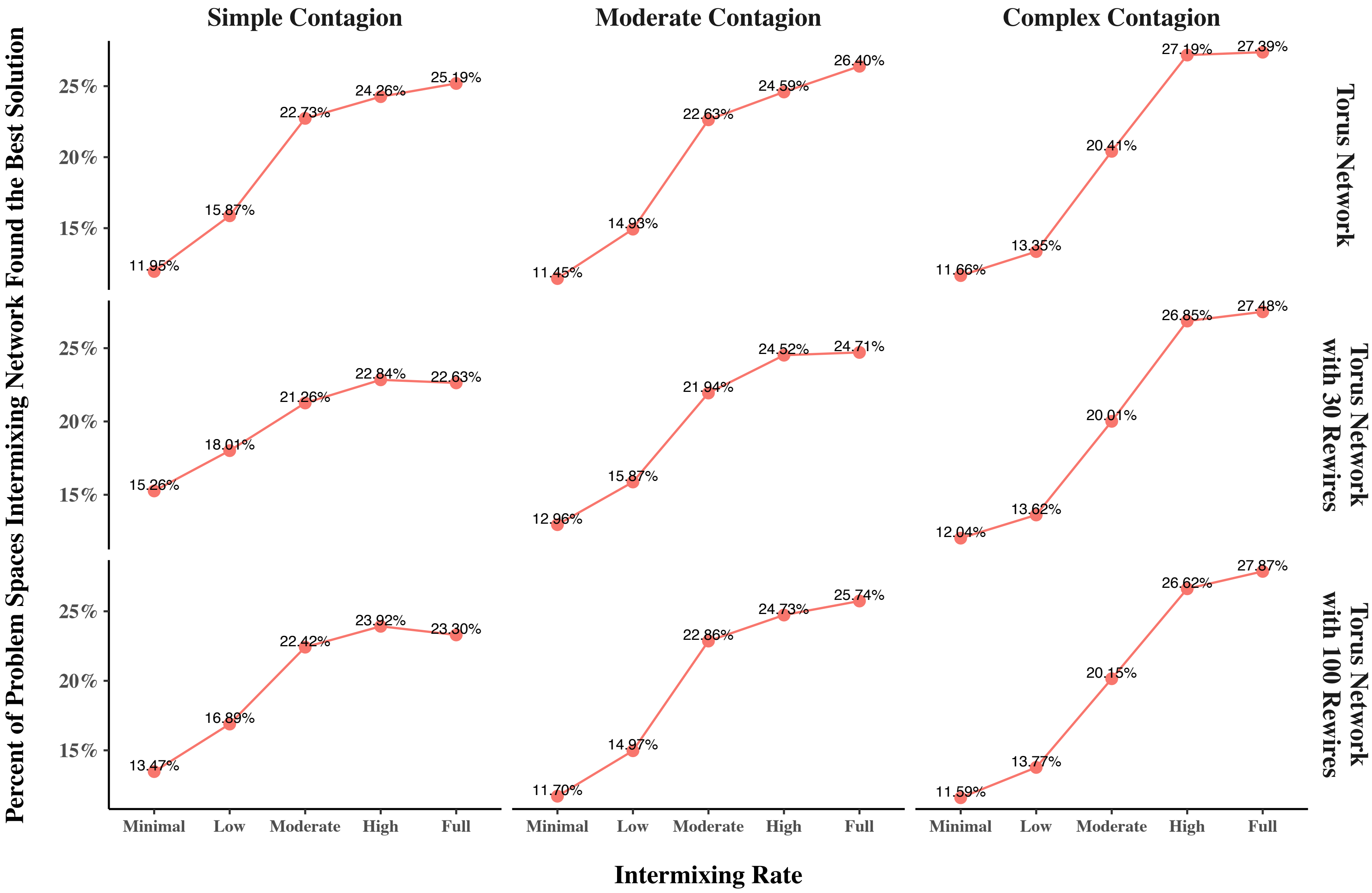

Supplementary Figure-7. Performance of networks with various intermixing rates for the diversity of knowledge simulations.

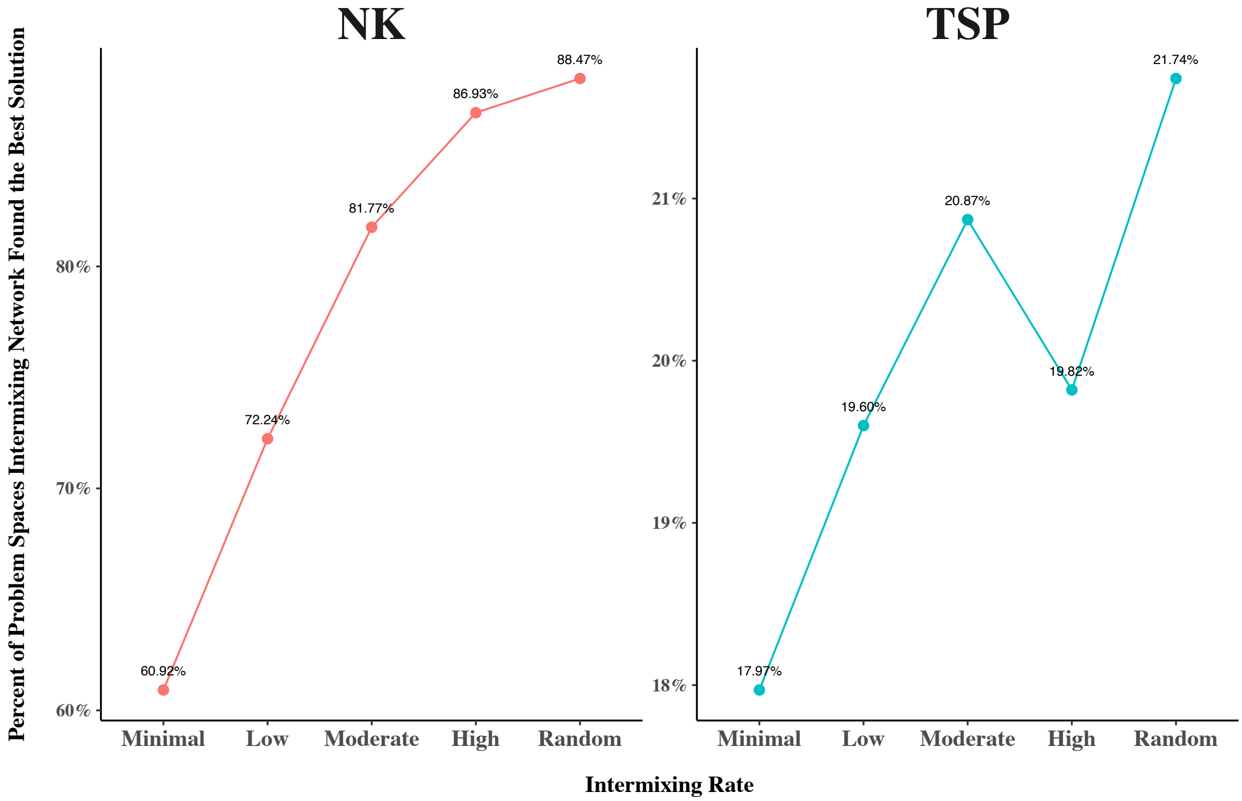

Supplementary Figure-8. Performance over time of networks with various intermixing rates for the diversity of knowledge simulations (NK and TSP spaces).

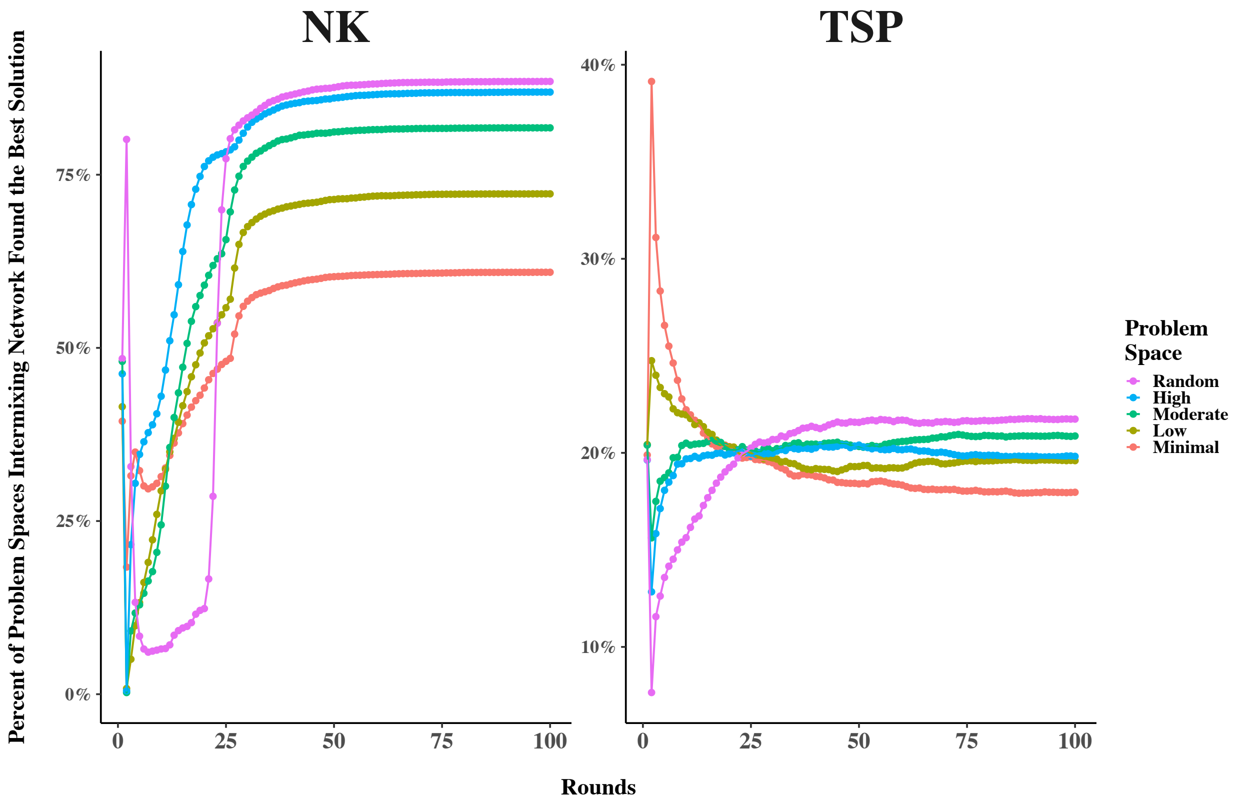

Supplementary Figure-9. Average number of unique states of knowledge with intermixing rates over time for the diversity of knowledge simulations (NK and TSP spaces). Error bars are the standard errors measured across NK and TSP spaces.

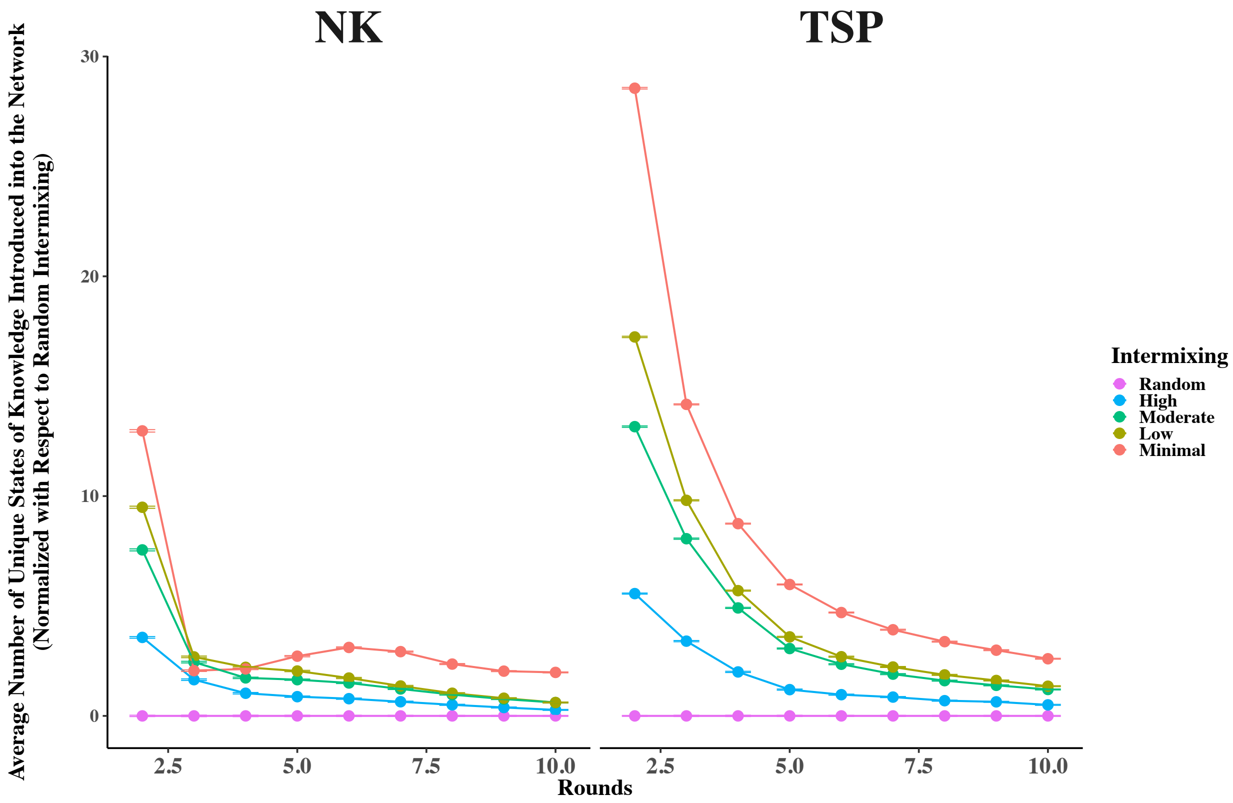

Supplementary Figure-10. Performance over time of networks with various intermixing rates for the diversity of knowledge simulations (small world and contagion setups, NK space)

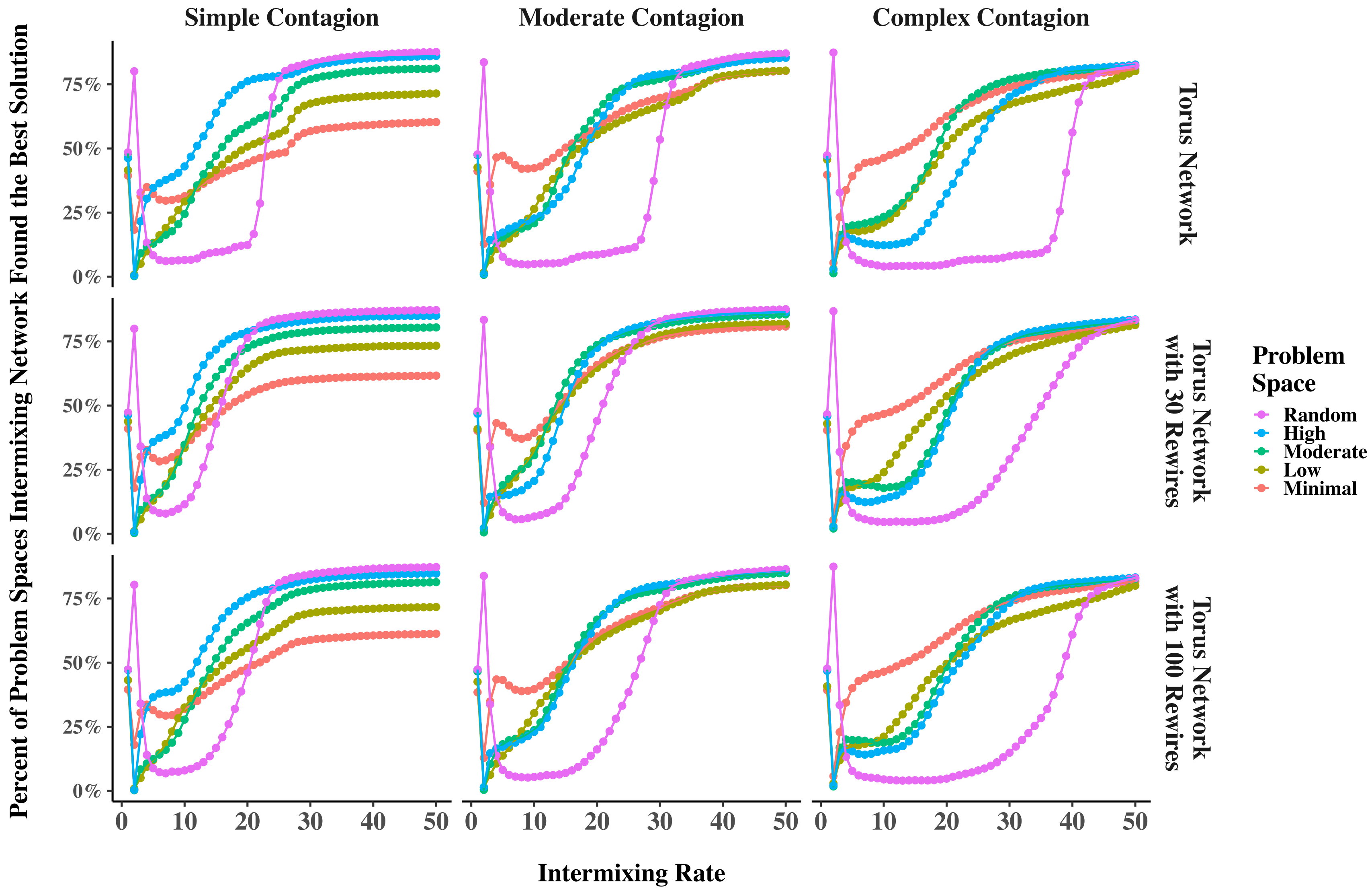

Supplementary Figure-11. Performance over time of networks with various intermixing rates for the diversity of knowledge simulations (small world and contagion setups, TSP space)

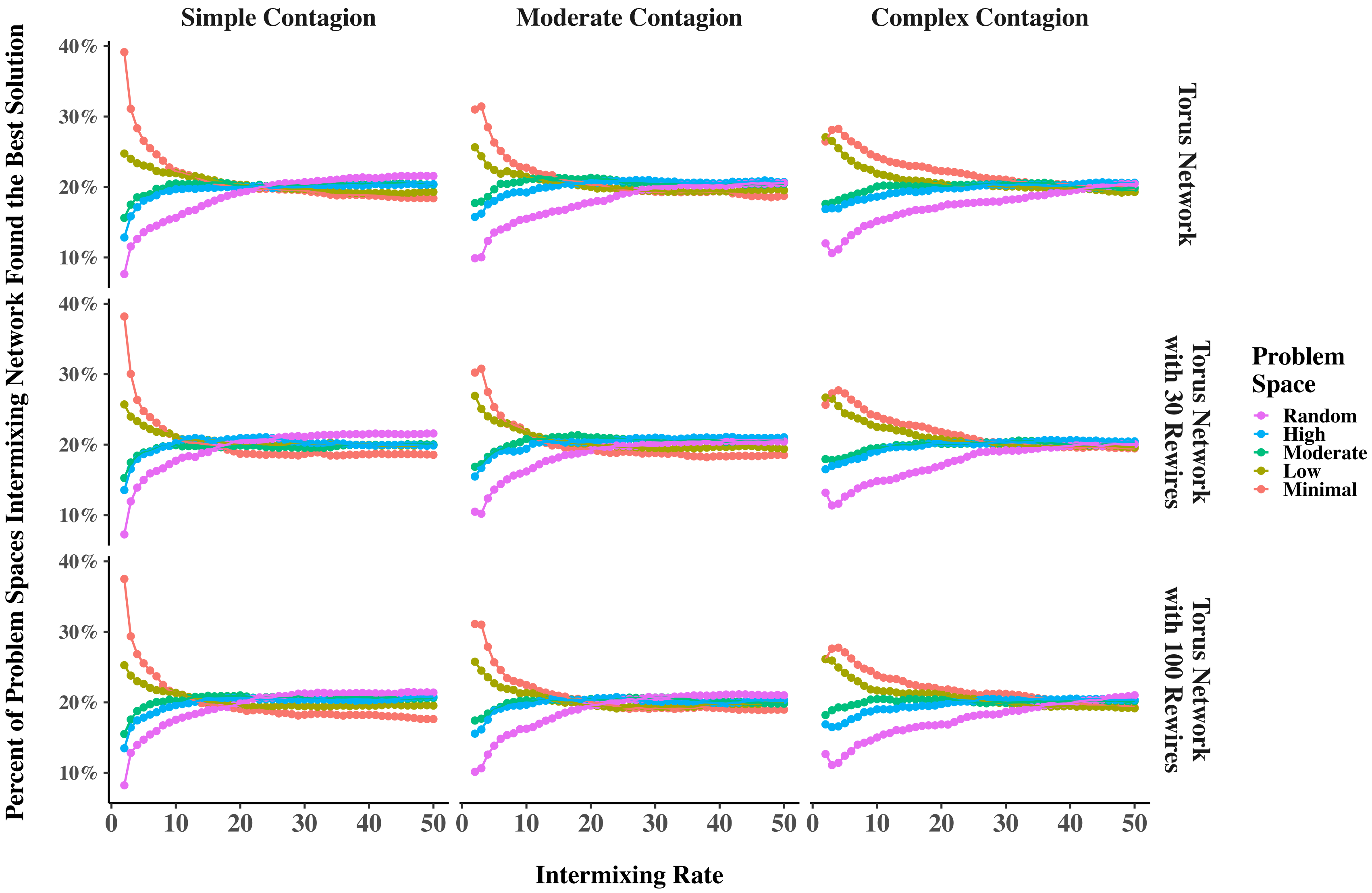

Supplement: Supplementary file 1 — Supplementary Information [file 41467_2019_12650_MOESM1_ESM.pdf]
